# Estimating Dining Plate Size From an Egocentric Image Sequence Without a Fiducial Marker

**DOI:** 10.3389/fnut.2020.519444

**Published:** 2021-01-14

**Authors:** Wenyan Jia, Zekun Wu, Yiqiu Ren, Shunxin Cao, Zhi-Hong Mao, Mingui Sun

**Affiliations:** ^1^Department of Electrical & Computer Engineering, University of Pittsburgh, Pittsburgh, PA, United States; ^2^Department of Bioengineering, University of Pittsburgh, Pittsburgh, PA, United States; ^3^Department of Neurosurgery, University of Pittsburgh, Pittsburgh, PA, United States

**Keywords:** wearable device, fiducial marker, dining plate size, egocentric image, technology-based dietary assessment

## Abstract

Despite the extreme importance of food intake in human health, it is currently difficult to conduct an objective dietary assessment without individuals' self-report. In recent years, a passive method utilizing a wearable electronic device has emerged. This device acquires food images automatically during the eating process. These images are then analyzed to estimate intakes of calories and nutrients, assisted by advanced computational algorithms. Although this passive method is highly desirable, it has been thwarted by the requirement of a fiducial marker which must be present in the image for a scale reference. The importance of this scale reference is analogous to the importance of the scale bar in a map which determines distances or areas in any geological region covered by the map. Likewise, the sizes or volumes of arbitrary foods on a dining table covered by an image cannot be determined without the scale reference. Currently, the fiducial marker (often a checkerboard card) serves as the scale reference which must be present on the table before taking pictures, requiring human efforts to carry, place and retrieve the fiducial marker manually. In this work, we demonstrate that the fiducial marker can be eliminated if an individual's dining location is fixed and a one-time calibration using a circular plate of known size is performed. When the individual uses another circular plate of an unknown size, our algorithm estimates its radius using the range of pre-calibrated distances between the camera and the plate from which the desired scale reference is determined automatically. Our comparative experiment indicates that the mean absolute percentage error of the proposed estimation method is ~10.73%. Although this error is larger than that of the manual method of 6.68% using a fiducial marker on the table, the new method has a distinctive advantage of eliminating the manual procedure and automatically generating the scale reference.

## Introduction

**Figure 1:**
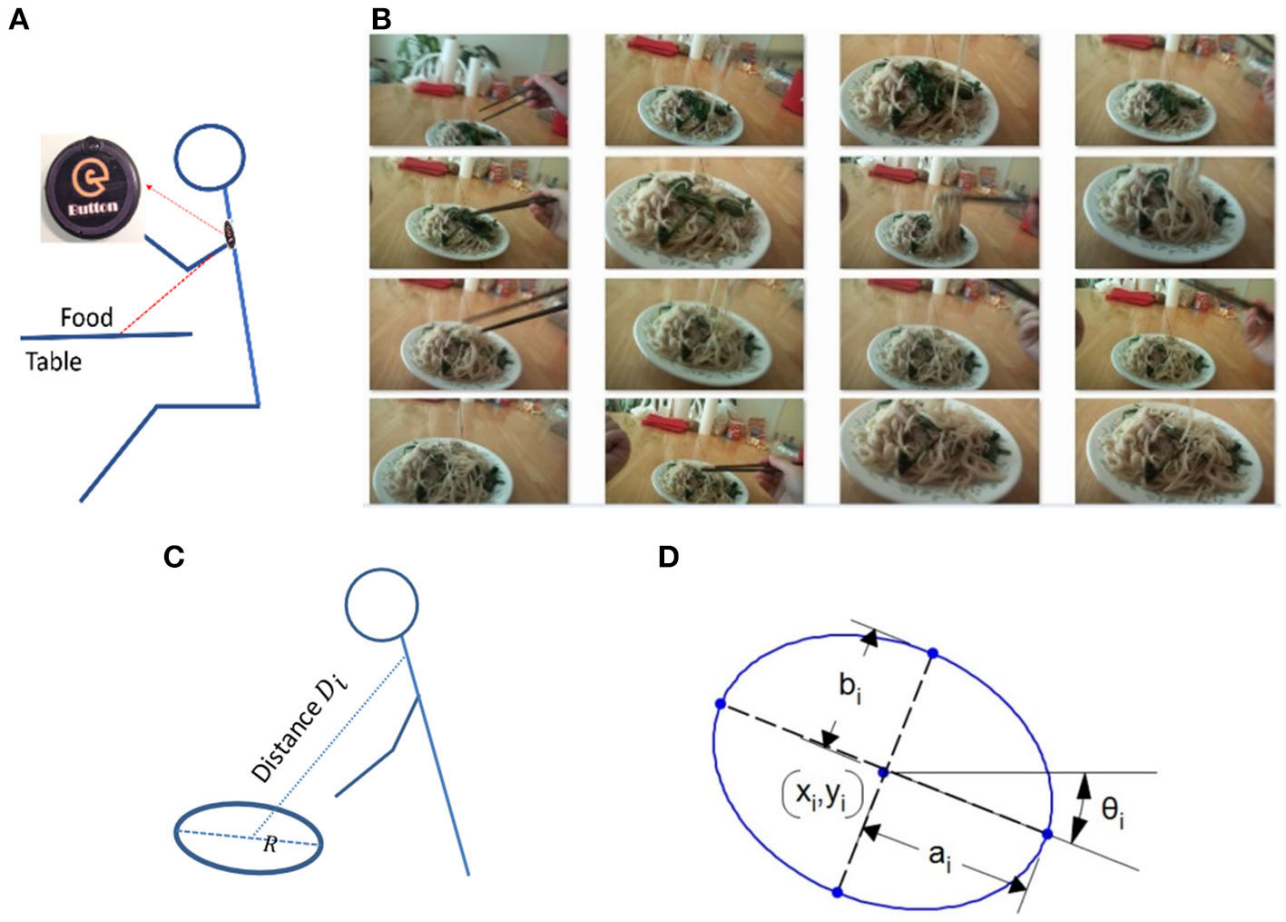
**(A)** A skeletal representation of a person wearing eButton during a meal; **(B)** Part of an egocentric image sequence acquired by the eButton showing quasiperiodic variations of the ellipses of the plate; **(C)** Definition of *D*_*i*_ ; **(D)** Parameters of an ellipse.

Many chronic diseases, such as heart diseases, cancer and diabetes, are associated with unhealthy diet. A recent study by the Global Burden of Disease found that poor diet accounted for ~20% of adult deaths in 2017 (1). As diet-related health risks are high, it is important to conduct dietary assessment among individuals' with, or in an emerging stage of, chronic diseases. Traditionally, this assessment depends on individuals' self-report, which is subjective and often inaccurate (2). In recent years, as microelectronic and mobile technologies advance, image-based dietary assessment has emerged (3, 4). The images of food are acquired from an individual either actively or passively. In the active approach, the individual takes pictures of his/her food before and after each eating event (5). Although this method is inexpensive (because of the wide availability of the smartphone) and the image quality is high, picture-taking must be volitionally initiated, which depends on the individual's memory. In the passive approach, the individual is provided with a small electronic wearable device, such as the eButton in the form of a chest pin [Figure 1A, (6, 7)]. This device is equipped with a wide-angle camera aiming at the food on the table during the eating process. Rather than taking pictures manually, a sequence of images is acquired automatically at a pre-set rate (4–6 s between images). For a complete dietary assessment, the device can be activated for the entire day, producing a large amount of data saved on the device. Once the data are uploaded to a computer, they are first screened using the Artificial Intelligence (AI) technology (8). This screening automatically filters out all image segments not containing foods or beverages, both reducing the burden of data examination by human experts and mitigating the related privacy concerns. The AI approach also allows objective studies of snacking and a wide range of other diet-related activities, such as food shopping, storage, preparation, cooking, and post-eating events. This work is in the domain of the passive approach.

Although image-based dietary assessment has many advantages over the traditional self-report method, it requires a scale reference within each image. The scale reference is extremely important, analogous to the importance of the scale bar in a map which enables the determination of the distance between any two points on the map or the area of any geological region covered by the map. Likewise, the volumes of foods and beverages on a dining table in the scope of an image cannot be determined without the scale reference. Currently, the scale reference is provided by a fiducial marker which is an object of known dimensions, such as a checkerboard or a business card (2, 5, 9). This method requires the individual to physically carry the card, place it on the dining table before the eating process and retrieve it afterwards. Clearly, these tasks are inconvenient and contradicts the goal of passive dietary assessment. In order to eliminate these tasks, we previously developed a method to use the dining plate itself as the scale reference (10, 11). Since a circular plate appears in the image as an ellipse and the eccentricity of the ellipse depends on the viewing angle of the wearable device, the coordinate transformation between the image pixel coordinates and the world coordinates can be established, under the condition that the radius of the plate is known. Although this method eliminates the need to carry, place and retrieve the fiducial marker, it requires a measurement of the plate radius, which is still a manual procedure and a significant burden to the participant. Eliminating this manual procedure would lead to a true passive dietary assessment, removing the last bottleneck that undermines the passiveness. Because of the high importance of this problem, considerable effort has been spent by the research community, and several approaches have been reported, such as using two cameras for a stereo view (12), adopting a depth camera (13), and using a laser reference produced by an add-on device (14, 15). Although these solutions are effective, the extra power consumption, enlarged wearable device size and increased cost have hampered their practical utility.

Unfortunately, elimination of the manual procedure and automatic determinization of the scale reference based purely on image contents represent an extremely difficult problem. The theory of computer vision has indicated that it is impossible to estimate the real size of an object in a single 2D image without providing the scale information (16). However, we will show, in this work, that this theoretical constraint can be circumvented if we use a sequence of images as the input and meet the following assumptions: (1) the heights of the dining table and chair at each dining location are fixed, (2) a one-time calibration is performed at each dining location using a circular plate of known size, (3) the individual uses the same wearable device affixed at the same body location to capture images, and (4) one of the food containers on the dining table is a circular plate. Then, we show that the desired scale reference can be determined automatically from the circular plate. Here we point out that, as in the case of a map where the scale bar is applicable to all geological regions covered by the map, this scale reference, once obtained, is applicable to all foods, beverages and other objects on the dining table. As a result, their lengths and volumes can be estimated from the image.

The rest of the paper is organized as follows. Section Methods presents the details of our method including the concepts utilized, the formulation of the method, and the plate radius estimation procedure. Section Experimental Results summarizes the experimental data and analysis results. In section Discussion, several issues of this method are discussed. Finally, limitations and future work are described in section Limitation and Future Work and conclusions are drawn in section Conclusion.

## Methods

### System Design Concepts

In real life, most individuals follow a certain eating pattern. With exceptions of traveling or “eating out,” they usually use fixed locations to have meals, for example, the kitchen or dining room at home for breakfast and dinner, and the office desk, a cafeteria, or a favored restaurant for lunch. At each location, the heights of the dining table and chair are usually fixed. Additionally, when a wearable device is used for dietary assessment, the location of the wearable device is usually fixed also, such as the chest location of the eButton (Figure 1A). All these factors indicate that, during eating events, the imaging environment of the individual at each dining location does not change drastically regardless of the food served and utensils utilized.

Although, as indicated previously, the theory of computer vision prohibits the determination of plate radius from a single image alone without the scale information, the estimation becomes possible when a sequence of images is captured by a wearable camera. Our key approach is to investigate the variation in the size of the observed dining plate in the image sequence (see an example in Figure 1B) as the result of the individual's repeated motion for reaching and fetching food. Although this body motion is not truly periodic (hence we call it “quasiperiodic motion”) involving considerable irregularities in the camera-to-plate distance, it is reasonable to assume that the statistical range of camera-to-plate distance variations remains the same for all eating events if the eating environment is fixed. From our previous studies (10), we know that the camera-to-plate distance can be calculated when a circular plate presents in the image and the plate size is known. If a one-time calibration with a plate of known size is conducted for an individual, the range of camera-to-plate distances during all future eating events of this person can be considered known. Then, the radius of an unknown plate can be estimated using this known range of the distances if his/her eating happens at the same location. These represent the key concepts of our method.

Our method, to be detailed below, for estimating the radius of an unknown plate from the image sequence is highlighted as follows. First, the relationship between the image of the plate (i.e., an ellipse) and the camera-to-plate distance is investigated and simplified. Then, a set of lines is generated to represent such relationship for different plate sizes. Next, from these lines, a particular line (i.e., the optimal line) is determined that best-matches the known range of the camera-to-plate distances obtained during the calibration process. The radius of the unknown plate is determined to be the radius represented by that line.

### Modeling Camera-to-Plate Distance

**Figure 2:**
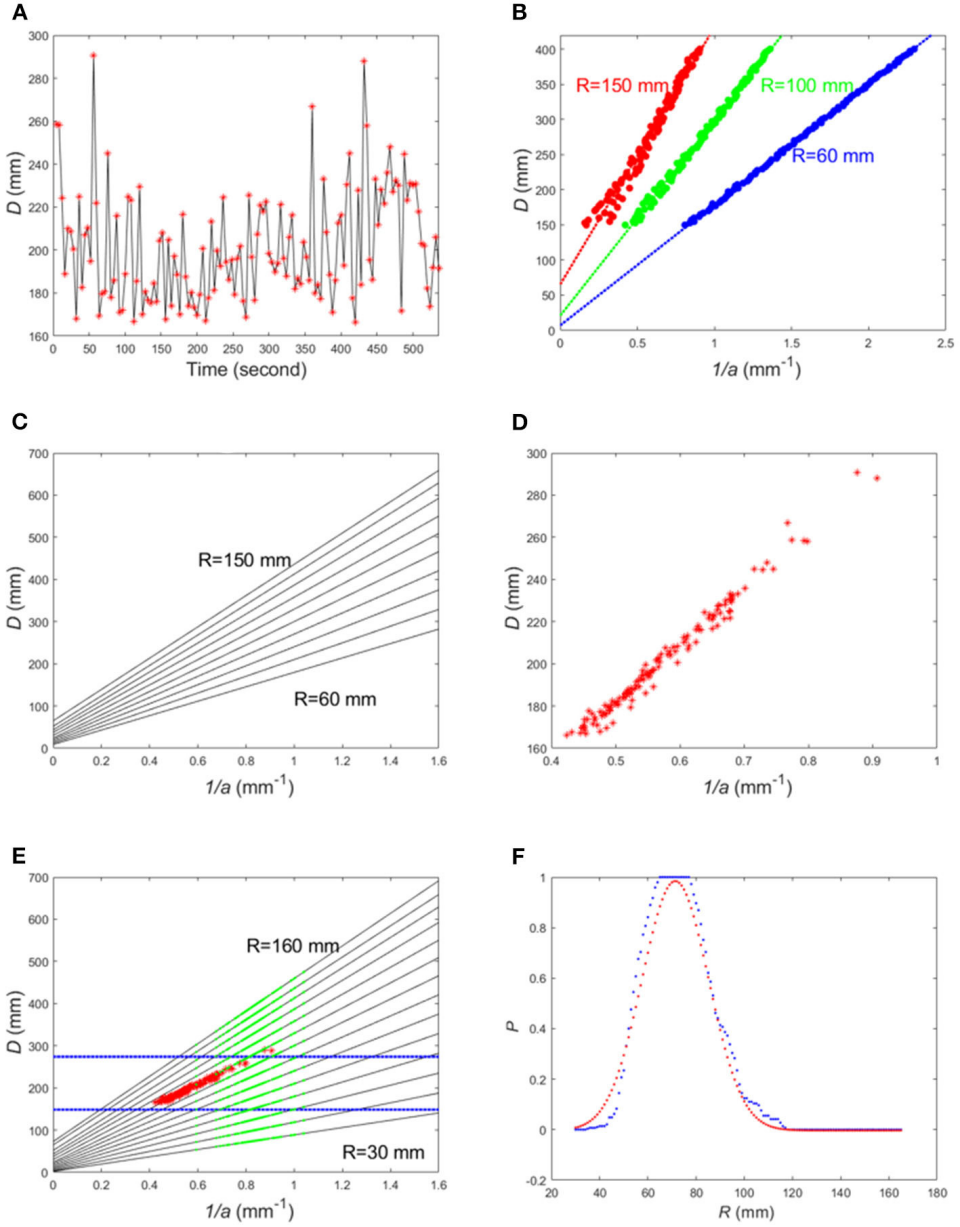
**(A)** Plot of change of camera-to-plate distance (red dots) during an eating episode; **(B)** Simulated camera-to-plate distance vs. 1/*a* using Equation (3); **(C)** A set of fitted curves for *R* = 60, 70, ⋯, 150 mm; **(D)** Camera-to-plate distance *D* vs. 1/*a* plot for the real data in **(A)**; **(E)** The two dotted blue lines represent the distance range [*D*_*l*_, *D*_*u*_] obtained from calibrated data (red dots). The black lines represent the fitted lines for different plate radii. The green dots are the corresponding *D*_*i*_ to each 1/*a*_*i*_ obtained from the image with unknown plate. **(F)** Plot of *P* and fitted Gaussian function.

Let *D*_*i*_ be the distance (unit: mm) between the lens of the wearable device to the center of the plate, where subscript *i* denotes the *i*^th^ image in the image sequence (Figure 1C). We have previously shown (10) that *D*_*i*_ can be determined from the ellipse (representing the plate) in the image if the actual radius of the plate is measured, and the intrinsic parameters of the camera, including the focal length and pixel size of the semiconductor chip, are provided. Figure 2A illustrates the change of camera-to-plate distance (red dots) during an eating episode. The mathematical expression for *D*_*i*_ is derived based on intersecting a cone (with its vertex located at the optical center of the camera) by the surface of the tabletop, where the circular plate (assuming that its height can be ignored) coincides with the intersection contour (10, 17). While the mathematical details of the expression are quite complex, here we write it as *g*, given by

(1) $$\begin{array}{l} D_{i}=g\left(x_{i},y_{i},\;a_{i},b_{i},\theta_{i},R\right), \end{array}$$

where (*x*_*i*_, *y*_*i*_) denote the coordinate of the center for the ellipse in the image; (*a*_*i*_, *b*_*i*_, θ_*i*_) represent the length of the semimajor axis, the semiminor axis, and the major axis angle of the ellipse, respectively (shown in Figure 1D); and *R* is the radius of the plate (unit: mm). Among the six variables of *g*, *R* is the only one that has a physical size in the world coordinates. With the ellipse parameters, the orientation and location of the dining table where the plate is placed on can be determined.

### Model Simplification

To simplify Equation (1) and make the relationship between *D* and (*x, y, a, b*, θ, *R*) more intuitive, we start with a simple case assuming that the optical axis of the camera goes through the center of the plate and the camera is level (the bottom of the camera is parallel to the horizon) but tilting downwards by an angle γ to capture the food on table. Under these assumptions, we explicitly derive function *g* based on a pin-hole camera model. Even with these simplifications, the derivation is still complex. It is thus not included here. Interested readers are referred to the Supplementary Material (attached). The final relationship between camera-to-plate distance *D* and the reciprocal of semimajor axis *a* of the observed ellipse is given by:

(2) $$\begin{array}{l} \frac{1}{a}=\frac{1}{f}\sqrt{{\left(\frac{D}{R}\right)}^{2}-\text{co}{\text{s}}^{2}\gamma }, \end{array}$$

where *R* is the plate radius, γ is the tilting angle, and *f* is the camera's focal length. The reason that the semiminor axis *b* is not included is also discussed in the Supplementary Material. Note that the unit of *a* is millimeter in the image plane (i.e., the sensor chip) within the camera. The conversion between the pixel coordinates in the image and the real-world coordinates in the image plane can be made through the intrinsic parameters of the camera (such as focal length, pixel size) (16).

With Equation (2), we can find the relationship between 1/*a* and *D* when *R* and γ are given. In practice, angle γ changes during eating due to human body's movement for reaching, fetching and delivering the food to the mouth (exemplified in Figure 1B as snapshots of this process). Thus, γ is set to a uniformly distributed random number between 20 and 70°. By simulation, a large number of pairs of (1/*a*_*i*_, *D*_*i*_), *i* = 1, 2, ⋯, *N*, using Equation (2) can be generated for different *R* values (see examples in Figure 2B). The red dots represent the data points for *R* = 150 mm, green dots for *R* = 100 mm and blue dots for *R* = 60 mm. It can be seen that the relationship between 1/*a* and *D* can be approximated by a linear function. By least-square fitting of the simulated data points for each *R*_*t*_ according to the following criterion

(3) $$\begin{array}{l} \underset{m,n}{\text{min}}\;\overset{N}{\underset{i=1}{\sum }}\;{\left(D_{i}-\frac{m}{a_{i}}-n\right)}^{2}, \end{array}$$

the fitting parameters *m, n* corresponding to the given radius *R*_*t*_ (*t* = 1, …, *T*) can be obtained, as shown in Figure 2B. Here *T* is the total number of simulated fitting lines. Then, we have

(4) $$\begin{array}{l} D_{i}\approx \frac{m}{a_{i}}+n\;\left(i=1,\ldots ,N\right). \end{array}$$

Figure 2C illustrates a case for *T* = 10 where each line represents a different value of *R*. Thus, for each *R* value, the ellipse parameter 1/*a* can be calculated from camera-to-plate distance *D*. Conversely, if *D* is known, we can determine *R*. Although *D* varies during the eating process as stated previously, the range of *D* is known from pre-calibration. If the calibrated range of *D* is [*D*_*l*_, *D*_*u*_] and the extracted ellipse parameters from the image sequence are {1/*a*_*i*_}, for *i* = 1, …, *N*, the problem of estimating the unknown plate size becomes finding the optimal line among all the simulated (or pre-tabulated) lines that best-maps the set of {1/*a*_*i*_} into the range of [*D*_*l*_, *D*_*u*_].

Although, in this simplification, the requirement that the optical axis of the camera goes through the plate center cannot be met normally, our data indicate that the approximate linear relationship between 1/*a* and camera-to-plate distance *D* still hold for real image sequences obtained during eating events (exemplified in Figure 2D). This demonstrates that the simplified model is generally acceptable. In some cases, however, the quasiperiodic body movement of the individual during eating is interrupted because of certain activities related or unrelated to the eating process (e.g., reaching a can of drink far away from the individual or operating a TV remote control). These activities result in sudden large changes in the positions and/or orientations in the observed sequence of ellipses. These changes do not fit our model but can be easily identified from the image sequence and discarded as data outliers.

### System Calibration and Plate Radius Estimation

In the following, we will first describe the calibration procedure. Then, we will provide two different estimates for the camera-to-plate distance, one by analytic calculation and the other by simulation. Finally, these two estimates are combined to estimate the unknown plate radius based on the result of calibration.

#### Calibration Procedure

A one-time calibration is required for each subject at each eating location. This calibration is nothing more than having a meal by the individual at the location with a circular plate of known radius *R*. From the calibration image sequence, the ellipse parameters are extracted from the *i*^th^ image and thus the camera-to-plate distance can be computed using Equation (1) which specifies the relationship between *D*_*i*_ and ellipse parameters. Although the mathematical expression for Equation (1) is complex, an analytic solution has been reported and can be computed using ellipse parameters (10, 17). From the whole image sequence, we can obtain a set of {*D*_*i*_} and a set of ellipse parameters {*x*_*i*_, *y*_*i*_, *a*_*i*_, *b*_*i*_, θ_*i*_}. Thus, the range of *D*_*i*_, defined as [*D*_*l*_, *D*_*u*_], can be estimated from the distribution of {*D*_*i*_}. Due to the limited number of images in a sequence and the noisy nature of the experimental data, the minimum and maximum value of *D*_*i*_, *i* = 1, 2, ⋯, *N*, may not reflect the actual distance range. We thus manipulate the histogram of {*D*_*i*_} to obtain the distance range, which will be described in section Data Analysis.

#### Camera-to-Plate Distance by Simulation

For a new image sequence including an unknown plate, the ellipse parameters (*x*_*i*_, *y*_*i*_, *a*_*i*_, *b*_*i*_, θ_*i*_) can also be extracted for each image in the sequence. Then, we set *R* to be a variable and equally sample this variable to form *R*_*t*_, *t* = 1, 2, ⋯, *T* with a sufficient range and resolution (e.g., from *R*_1_= 30 mm to *R*_*T*_= 165 mm with an increment of 1 mm). Next, the fitting parameters *m, n* corresponding to the given radius *R*_*t*_ (*t* = 1, 2, …, *T*) are obtained using Equation (3) as illustrated in Figure 2B. By substituting *a*_*i*_ to the simplified form of Equation (1), i.e., Equation (4), the camera-to-plate distances *D*_*i*_, *i* = 1, 2, ⋯, *N*, for each *R*_*t*_, *t* = 1, 2, ⋯, *T*, can be simulated (i.e., pre-tabulated), defined as $\left\{D_{i}^{1}\right\}.$

#### Calculated Camera-to-Plate Distance

Since the available data obtained from an eating event are usually limited *(N* is usually <100), we calculate another set of $\;\left\{D_{i}\right\}\text{ called }\left\{D_{i}^{2}\right\}\;$ for the same values of *R*_*t*_, *t* = 1, 2, ⋯, *T*, using Equation (1) although the calculation is complicated (10, 17). The main reason of adding this part of calculation is to double the number of data points that can be used to make the estimation more reliable.

#### Plate Size Estimation

$\text{After combining the two sets of }\left\{D_{i}\right\}\text{ as }\left\{D_{i}\right\}=\left\{D_{i}^{1}\right\}\cup \left\{D_{i}^{2}\right\}$ for each *R*, the number of {*D*_*i*_} that fall into the calibrated range [*D*_*l*_, *D*_*u*_] can be counted. An index representing how close each *R* is to the actual radius can be calculated as *P* = |{*D*_*i*_ ∈ [*D*_*l*_, *D*_*u*_]}|/|{*D*_*i*_}| (see Figure 2F for an example), where the vertical bars “|·|” represent the number of elements in a set. Finally, we fit the curve with a Gaussian function, and the estimated *R* corresponds to this maximum point (i.e., the mean of the Gaussian distribution).

## Experimental Results

To validate our plate radius estimation method, we conducted experiments in real-world settings. In this section, we describe the details of our experiments, including human subjects, experimental procedure, data analysis, and experimental results.

### Human Subjects

With an approval by the Institutional Review Board at the University of Pittsburgh, three human subjects participated in the experimental study. In order to satisfy the assumptions presented in section Model Simplification, these subjects were selected based on the following criteria: (1) they were healthy with normal body posture at both sitting and standing positions; (2) they followed a regular daily routine during the study (e.g., traveling was excluded); and (3) their dining locations were mostly fixed.

### Experimental Procedure

The subjects were first trained for using the eButton to record their dining events. They were instructed to comply with the following requirements: (1) using circular plates as the food container for serving; (2) wearing the eButton at a fixed chest location; and (3) keeping the heights of dining table and chair at each dining location unchanged. The subjects were instructed to follow their regular dietary patterns without restrictions on types of food and activities while sitting at the table (e.g., listening to music, watching TV, making a phone call, or interacting with people). No limitation was imposed on food types and utensils.

In each meal during the experiment, the subject wore the eButton and had meals normally using the pre-measured plate. The measured values were used either for the calibration process or as the gold standard for assessing the accuracy of our plate radius estimation algorithm.

### Data Analysis

After the study, the subjects returned the eButton to our laboratory where the recorded data were read from the microSD card within the device. The following data analysis steps were implemented.

#### Image Screening and Ellipse Extraction

All the images in each eating event were visually examined by a researcher. The images that contained no plate or a plate with most of its boundary missing were regarded as outliers and excluded from data analysis. For each image, the contour of the plate edge, observed as an ellipse in the image, was first extracted automatically using an automatic algorithm developed by us previously (18). In some cases, the automatic method failed due to occlusion or shadowing. In these cases, we used interactive method in which six points on the ellipse were manually selected. In either case, the parameters of each ellipse (e.g., semimajor axis *a*) were extracted by a least-squares fitting of the ellipse boundary.

#### Distance Range From Calibrated Image Sequence

For each image in the calibrated image sequence, all {*x*_*i*_, *y*_*i*_, *a*_*i*_, *b*_*i*_, θ_*i*_}, *i* = 1, 2, ⋯, *N*, where *N* is the number of images in an eating event after eliminating outliers, were extracted. Then, distance *D*_*i*_ corresponding to each image was obtained using Equation (1) with the pre-measured *R*. The red dots in Figures 2D,E represent the pairs of {1/*a*_*i*_, *D*_*i*_} calculated from the calibrated image sequence. To determine the range of {*D*_*i*_} reliably, the histogram of {*D*_*i*_} was calculated and the values in the two extreme bins were removed if the frequency in either bin was small (i.e., less than half of the average frequency). After that, the maximum and minimum values of the remaining *D*_*i*_ were set to [*D*_*l*_, *D*_*u*_]. Examples are shown in Figure 2E.

#### Simulation of the Relationship Between Ellipse Parameter and Camera-to-Plate Distance

The simulation was described in section System Calibration and Plate Radius Estimation. Simulated lines represent the relationship between *D* and 1/*a*, as shown in Figure 2C. In our experiment, the range of was chosen from 30 to 165 mm with an increment of 1 mm.

#### Plate Radius Estimation From the Image Sequence With Unknown Plate Size

For each human subject at each dining location, we collected data containing different eating events using plates of different radii. We call this collection of data “eating episodes” in which each episode is a particular event in the collected dataset. We took each plate as the calibration/reference plate sequentially from the dataset and the radii of the remaining plates were estimated using the procedure described in section System Calibration and Plate Radius Estimation. Our experiment resulted in *M*(*M* − 1) estimates of plate radii for each human subject where *M* is the number of plates utilized by the subject during the experiment.

#### Statistical Analysis

To observe the estimation error statistically, we calculated the percentage error for the estimated plate radius in each eating episode using different plates for calibration. Then, we calculated several statistical measures, including the mean Percentage Error (mPE), mean absolute Percentage Error (maPE), mean relative Root Mean Square Error (mrRMSE), defined as follows:

$$\begin{array}{l} Percentage\;Error\;\left(PE\right)=\frac{R_{k,j}-R_{j}}{R_{j}} \end{array}$$

$$\begin{array}{l} mean\;Percentage\;Error\left(mPE\right)=\;\frac{1}{M\left(M-1\right)}\underset{k}{\sum }\underset{j\neq k}{\sum }\frac{R_{k,j}-R_{j}}{R_{j}} \end{array}$$

$$\begin{array}{l} mean\;absolute\;Percentage\;Error\;\left(maPE\right)\\ \;=\;\frac{1}{M\left(M-1\right)}\underset{k}{\sum }\underset{j\neq k}{\sum }\left|\frac{R_{k,j}-R_{j}}{R_{j}}\right| \end{array}$$

$$\begin{array}{l} mean\;relative\;Root\;Mean\;Square\;Error\;\left(mrRMSE\right)\\ \;=\sqrt{\frac{1}{M\left(M-1\right)}\underset{k}{\sum }\underset{j\neq k}{\sum }{\left(\frac{R_{k,j}-R_{j}}{R_{j}}\right)}^{2}} \end{array}$$

where *R*_*j*_ is the true radius of the plate in the *j*th episode, *R*_*k,j*_ is the estimated radius of plate in the *j*th eating episode using the plate in the *k*th episode as the reference plate for calibration, and *M* is the total number of episodes. Note that each error calculation is represented in the percentage value.

### Results

**Figure 3:**
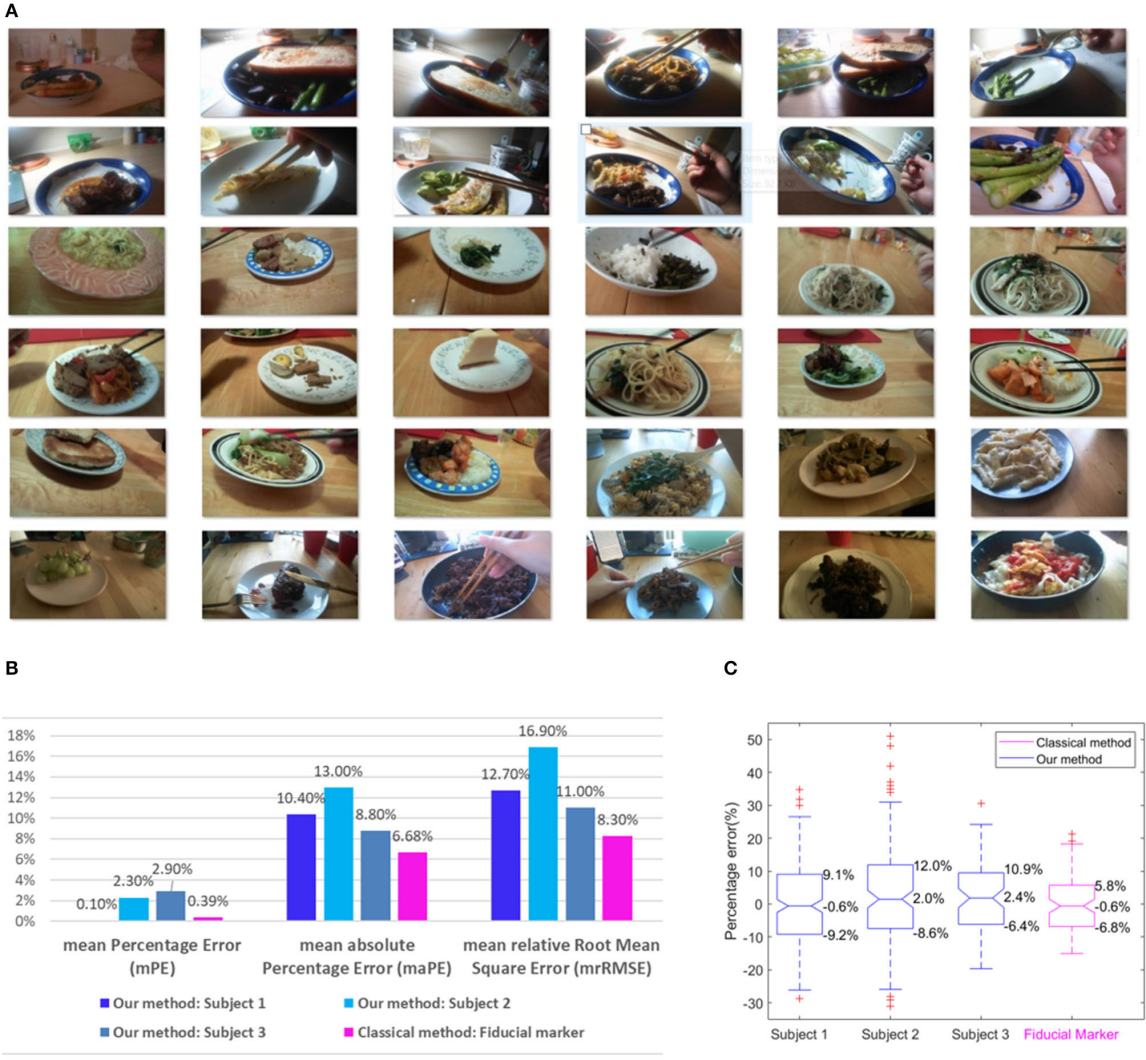
**(A)** Illustrations of one typical image for each episode; **(B)** Calculated mPE, maPE, and mrRMSE for each subject and the fiducial marker method; **(C)** Box-plot of percentage errors for each subject with extreme percentage errors, 25th percentiles of errors, median, and 75th percentiles of errors marked for the three subjects and the fiducial marker method.

In our experiments, a total of 37 eating episodes (15 for Subject 1, 12 for Subject 2, and 10 for Subject 3) were recorded, and the plate radius used in each episode was measured as the ground truth. One episode of Subject 3 was removed from further analysis because the number of images in this episode was insufficient. Thus, 36 episodes were analyzed, and 15 different circular plates with different radii and heights were used in this study. Typical images are illustrated in Figure 3A, where one image is shown for each episode. In these eating episodes, the foods consumed included beef, rice, noodle, dissert, bread, Chinese pancake, pasta, and different kinds of vegetables. Chopsticks, forks, knifes, and spoons were used as utensils.

While our results for all subjects are summarized in Figure 3, specific values of estimated plate radii for Subject 3 are provided in Table 1 as an example. Total nine tests were conducted for this subject. In this table, the values in each row (denoted by “Test #”) represent estimated radii of different plates using the same reference, while the values in each column (denoted by “Plate #”) represent estimated radii of the same plate using different references. The boldfaced values along the diagonal lines are true radii, which are actually measured values. The calculated mPE, maPE, mrRMSE using the formula in section Data Analysis for each subject are listed in Figure 3B. A set of statistical measures is provided in Figure 3C, including distributions of percentage errors for all subjects, 25th percentiles of errors, 75th percentiles of errors, and median errors.

**Table 1:** Comparison of measured (ground truth) and estimated plate radii for Subject 3.

	**Plate#1**	**Plate#2**	**Plate#3**	**Plate#4**	**Plate#5**	**Plate#6**	**Plate#7**	**Plate#8**	**Plate#9**
Test#1	**130**	131	107	120	97	100	133	104	98
Test#2	130	**125**	103	115	93	96	128	99	95
Test#3	121	117	**95**	108	88	90	120	93	89
Test#4	148	146	118	**127**	107	110	147	114	109
Test#5	141	135	111	124	**100**	103	139	107	102
Test#6	154	153	124	141	113	**112**	154	121	115
Test#7	139	135	109	123	100	102	**130**	106	101
Test#8	132	128	105	118	95	98	131	**100**	97
Test#9	130	126	103	116	94	96	129	100	**95**

*The numbers in each row (Test #) represent estimated radii of different plates using the same reference, while the numbers in each column (Plate #) represent estimated radii of the same plate using different references. The boldfaced numbers on the diagonal lines are true radii (actually measured values)*.

In order to compare the accuracies of our automatic and the traditional manual methods, we conducted an additional experiment using a fiducial marker, which was a rectangular checkerboard of 6 × 7 cm. Ten circular plates with different radii and heights were utilized in this comparative experiment. The range of the plate radii was identical to that in the previous experiment. The checkerboard card was manually placed next to each plate before taking pictures with an eButton. Since the thickness of checkerboard was small, its surface can be considered as the same surface of the table. Due to the plate height, the plane of table surface estimated from the checkerboard in the image was different from the plane of the plate border, causing a small amount of error in plate radius estimation. For a fair comparison with our method, we assumed that the plate height was standard, which was the height of the reference plate, the same as the assumption made in our method. Under this assumption, each of the ten plates was taken as the reference plate and the remaining nine plates were estimated. Thus, total 90 plate radius estimates were obtained. In each estimate, five images in different viewing distances and angles were processed, and the five results were averaged. Example pictures, the data processing algorithm, estimated values and estimation errors are included in the Supplementary Material. Finally, the estimation errors were studied using the same statistical measures (i.e., mPE, maPE, mrRMSE, and boxplot), as in the previous experiment. The results of this comparative experiment are summarized in Figures 3B,C. It can be observed that our automatic method has a larger error than the manual method using a fiducial marker (10.73% vs. 6.68% in terms of the mean absolute percentage error). This is not surprising since the fiducial marker provides a scale reference directly in the image. Although a larger error is involved, the new method has a distinctive advantage of eliminating the manual procedure and automatically generating the scale reference.

## Discussions

In this work, we develop a new method to eliminate the requirement for a fiducial marker in egocentric image based dietary assessment. We take advantage of the fixed environment at the dining location to model the eating behavior of an individual. Our study yields a new method to estimate the dining plate radius automatically. If there is only one plate of food in the image, the plate radius (or diameter) is sufficient to serve as the scale reference. In cases where the captured image shows multiple foods on the table, we need to go only one step further. Using this radius and the orientation information obtained from the observed elliptic shape of the plate, a plane equation for the tabletop can be determined which serves as the desired scale reference. This plane equation is easy to obtain because the ellipse in the image provides the orientation (or the norm vector) of the plate, the circle of the plate is in or close to the plane of the tabletop, and the radius provides the scale in a real-world unit (e.g., mm). Analogous to a map where the sizes of all regions in the map can be estimated using the scale bar, the sizes or volumes of any foods (within containers of any forms or shapes or even without containers) or beverages on the table can be estimated using the scale reference. Compared with the existing methods using additional sensors and laser emitters, our method requires no added cost. A simple, once-for-all calibration is the only requirement to implement our method.

Our method is built upon a number of assumptions: (1) it is applicable only to each individual, (2) the heights of the dining table and chair at the dining location are invariant, (3) the device-wearing position on the body is fixed, and (4) the range of body rotation during normal eating is invariant. Clearly, these are strong assumptions which may not be met exactly in a real-world setting. However, making such assumptions is a key step to simplify the complex six-variable relationship (Equation 1) into a single-variable linear equation (Equation 4). Our experimental results have indicated that, even if the assumptions are not met completely, the mean absolute percentage error of plate radius estimation is <11%. Nevertheless, attention should be paid to the validity of the data as we did in data analysis. It is strongly recommended to exclude the images with a considerable portion of the plate shifted out of the image frame. These cases can be easily identified from the image data.

In our method, estimating the range of the distance from the calibrated image sequence is an important step. However, due to the limited data points in an image sequence (e.g., the eButton acquires one image in every 4–6 s, preset by the user), the estimation of the distance range may not be sufficiently accurate. Increasing the frame rate of the wearable camera to obtain more images may improve the estimation.

We would also like to point out two main reasons of using a circular plate to obtain the scale reference. First, it is a commonly used utensil in most parts of the world. Second, if the plate is shallow, its top surface is close and parallel to the table surface. However, with exception of the disposable paper plate, most plates have significant heights. In our algorithm, we implicitly assume that the height of the reference plate is the height of the unknown plate, and this “standard height” is used as an offset to be considered in the plane equation for the tabletop. Nevertheless, this method involves a certain error. In some cultures, bowls are used more commonly than plates. We point out that our method can still be used by changing the reference plate to the reference bowl and use its height as the standard height, with some tolerance of the height-related error. Finally, since our method relies only one circular plate to estimate the scale reference, in our experiments, each image contains only a single plate. However, our method is applicable to images containing multiple foods in any forms of containers as long as one of them is a circular plate (or bowl if the reference is a bowl).

The result of the comparative experiment indicates that the manual fiducial marker method is more accurate than our automatic method. This is understandable because the marker provides a scale reference directly while the automatic method does not have such information. However, in the fiducial marker method, a checkerboard card must be carried by the individual, placed on the tabletop next to the food before eating, and retrieved after eating for the next use. These procedures are unwelcome and can be forgotten easily.

## Limitations and Future Work

Our method provides an automatic way to estimate the size of a circular plate. Therefore, as long as there is a plate on the table and the assumptions about the fixed eating environment are satisfied, we will be able to obtain a scale reference for all items on the table based on a one-time calibration procedure. If there are bowls, glasses/cups and/or snacks placed on the same table, in theory, their volumes can be estimated based on the scale reference that our method provides. However, the estimation is subject to various constraints, assumptions and, in some cases, availability of a certain set of knowledge (e.g., the shape of a bowl or a cup). In addition, the problem of 3D food volume estimation from a single or a series of 2D images has not yet been fully solved, and there is a strong demand to develop new computational methods using advanced technologies, such as artificial intelligence (AI). Even though this volume estimation problem is fascinating, its discussion would be lengthy, beyond the scope of this paper which is focused solely on automatic plate radius estimation to generate a scale reference. We emphasize again that this reference is a fundamental requirement regardless the technologies to be utilized.

Our method is currently limited to the dining scenarios where a circular plate or bowl is used as a food container. For the cases where only non-circular plates or bowls are present in the image, we have not found an effective method to estimate their parameters without pre-measurements. These types of containers are still the subjects of further investigation.

## Conclusion

We have developed a new method to estimate the radius of a dining plate in a sequence of egocentric images acquired by a wearable device thus a scale reference can be obtained automatically. This method is based on mathematical analysis of the eating behavior of an individual and the invariance of the eating environment (i.e., the heights of the table and chair are fixed at each dining location). Unlike the traditional methods that use a fiducial marker or require measurement of plate radius for every meal, our method requires only a once-for-all radius measurement of a single plate. After this calibration step, the radius of arbitrary plate can be estimated. Due to the elimination of a fiducial marker, our method greatly reduces the research burden for research participants, making the dietary assessment passive and objective.

## Ethics Statement

The studies involving human participants were reviewed and approved by University of Pittsburgh Institutional Review Board. The patients/participants provided their written informed consent to participate in this study.

## Author Contributions

MS, ZW, and WJ were responsible for concept formulation and methodological design. ZW, YR, SC, and WJ conducted data collection and image processing. WJ, YR, ZW, Z-HM, and MS contributed to the algorithm for data analysis, final drafting, and editing of the manuscript. All authors contributed to the article and approved the submitted version.

## Conflict of Interest

The authors declare that the research was conducted in the absence of any commercial or financial relationships that could be construed as a potential conflict of interest.
