# Supplementary material for: Estimating Dining Plate Size From an Egocentric Image Sequence Without a Fiducial Marker
Source: Front Nutr. 2021 Jan 14;7:519444. doi: 10.3389/fnut.2020.519444 (PMC7840562; doi:10.3389/fnut.2020.519444)
Supplement: Supplementary file 1 [file Data_Sheet_1.docx]

**Supplementary Material**

## Supplements to Section 2.3: Modeling Camera-to-Plate Distance

When a circular feature (i.e., a plate) presented in an image and its radius is known, the distance and orientation of camera relative to the circular feature can be estimated (Safaee-Rad et al., 1992; Jia et al., 2012). The mathematical expression for the distance $D_{i},$defined as the distance between the camera optical center to the center of the plate, and the ellipse parameters of the circular feature in the image can be written as$g$, given by

$$D_{i}=g\left( x_{i},y_{i}, a_{i},b_{i},\theta_{i},R \right), \left( A.1 \right)$$

where ${(x}_{i},y_{i})$ denote the coordinate of the center for the ellipse in the image; $\left( a_{i},b_{i},\theta_{i} \right)$ represent the length of the semimajor axis, the semiminor axis, and the major axis angle of the ellipse; and $R$ is the radius of the plate (unit: mm).

To simplify Eq. (A.1) and make the relationship between *D* and $\left( x,y,a,b,\theta\right)$ intuitive, we start with a simple case with the assumption that the optical axis of the camera goes through the center of the plate and the camera is level but looking down at an tilting angle $\gamma$, shown in Figure A1.

From Figure A1, because $\Delta$ALO $\sim\Delta H$FO and $\Delta$CMO $\sim\Delta$GFO, the following equations can be obtained:

$\left\{ \begin{aligned} \frac{R\sin\gamma}{b_{1}} = \frac{D+R\cos\gamma}{f} \\ \frac{R\sin\gamma}{b_{2}} = \frac{D-R\cos\gamma}{f} \end{aligned} \right.$. (A.2)

Then

$\left\{ \begin{aligned} b_{1} = \frac{fR\sin\gamma}{D+R\cos\gamma} \\ b_{2}= \frac{fR\sin\gamma}{D-R\cos\gamma} \end{aligned} \right. .$ (A.3)

From Figure A1, we know

$b= \frac{b_{1} + b_{2}}{2}.$ (A.4)

Substituting (A.3) into (A.4), we have

$b= \frac{fR\sin\gamma}{2}(\frac{1}{D+R\cos\gamma}+ \frac{1}{D-R\cos\gamma})$ $= \frac{DfR\sin\gamma}{D^{2}- R^{2}\cos^{2}\gamma} .$ (A.5)

From Figure A1, we know

$w = \frac{b_{2}- b_{1}}{2} = \frac{fR\sin\gamma}{2}(\frac{1}{D-R\cos\gamma}- \frac{1}{D+R\cos\gamma})$ $= \frac{fR^{2}\sin\gamma\cos\gamma}{D^{2}-R^{2}\cos^{2}\gamma} .$ (A.6)

Similarly, considering $\Delta\mathrm{EK}$O and$\Delta\mathrm{FB}$O, we have

$\frac{f}{w}= \frac{D-v\cos\gamma}{v\sin\gamma}$ . (A.7)

Combine (A.6) and (A.7), we obtain

$\frac{D-v\cos\gamma}{v\sin\gamma} =\frac{D^{2}-R^{2}\cos^{2}\gamma}{R^{2}\sin\gamma\cos\gamma} .$ (A.8)

Thus

$v = \frac{R^{2}\cos\gamma}{D}$ . (A.9)

In $\Delta$EOK, we also have

$d= \sqrt{D^{2}+v^{2}-2Dv\cos\gamma}$ . (A.10)

Because$\Delta$PJO $\sim\Delta$QNO

$\frac{\sqrt{R^{2}-v^{2}}}{a}= \frac{d}{\sqrt{f^{2}+w^{2}}}$ . (A.11)

Substituting (A.6), (A.9) and (A.10) into (A.11), we obtain

$\frac{1}{a}= \frac{1}{\sqrt{f^{2}+w^{2}}} \frac{\sqrt{D^{2}+v^{2}-2Dv\cos\gamma}}{\sqrt{R^{2}-v^{2}}}$ $= \frac{1}{\sqrt{f^{2}+\left( \frac{fR^{2}\sin\gamma\cos\gamma}{D^{2}-R^{2}\cos^{2}\gamma} \right)^{2}}} \frac{\sqrt{D^{2}+\frac{R^{4}\cos^{2}\gamma}{D^{2}}-2D\frac{R^{2}\cos\gamma}{D}\cos\gamma}}{\sqrt{R^{2}-\frac{R^{4}\cos^{2}\gamma}{D^{2}}}}$

$=\frac{1}{f}\frac{1}{\sqrt{1+\left( \frac{R^{2}\sin\gamma\cos\gamma}{D^{2}-R^{2}\cos^{2}\gamma} \right)^{2}}} \frac{\sqrt{D^{4}+R^{4}\cos^{2}\gamma-2D^{2}R^{2}\cos^{2}\gamma}}{R\sqrt{D^{2}-R^{2}\cos^{2}\gamma}}$

=$\frac{1}{f}\frac{D^{2}-R^{2}\cos^{2}\gamma}{\sqrt{{(D^{2}-R^{2}\cos^{2}\gamma)}^{2}+{(R^{2}\sin\gamma\cos\gamma)}^{2}}}\frac{\sqrt{D^{4}+R^{4}\cos^{2}\gamma-2D^{2}R^{2}\cos^{2}\gamma}}{R\sqrt{D^{2}-R^{2}\cos^{2}\gamma}}$

$=\frac{1}{f}\frac{\sqrt{D^{2}-R^{2}\cos^{2}\gamma}}{R}$ =$\frac{1}{f}\sqrt{\left( \frac{D}{R} \right)^{2}-\cos^{2}\gamma} .$ (A.12)


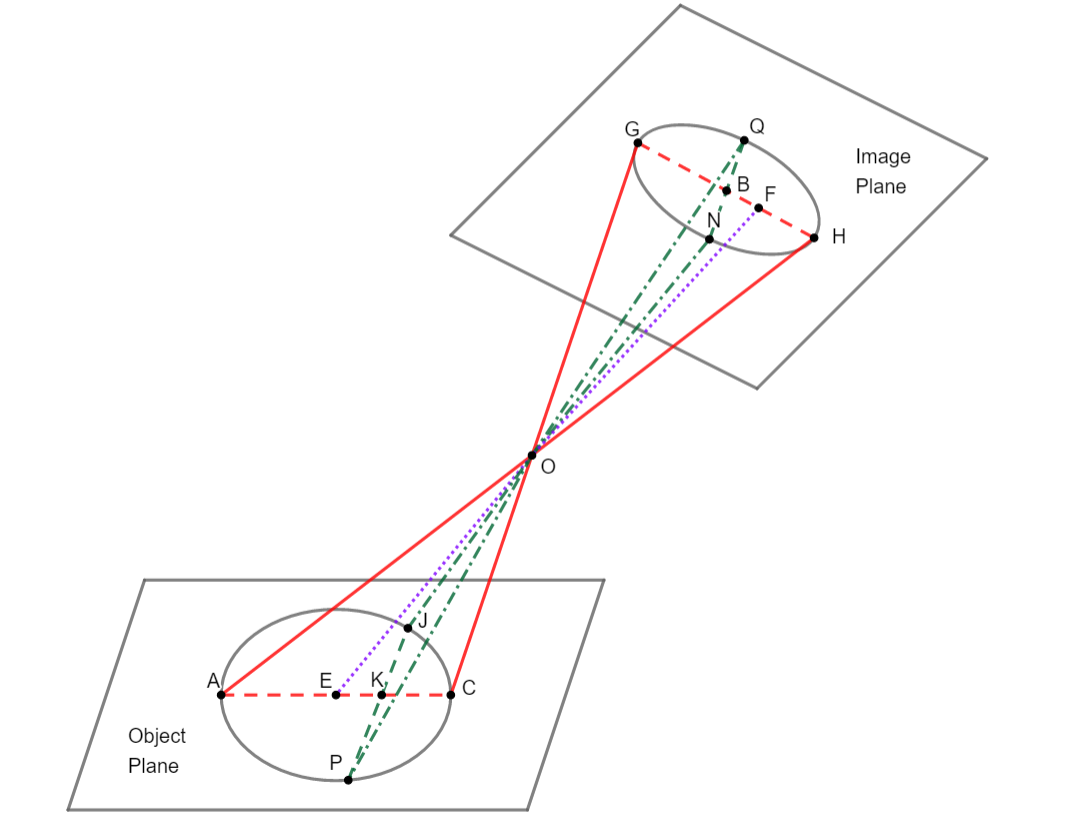


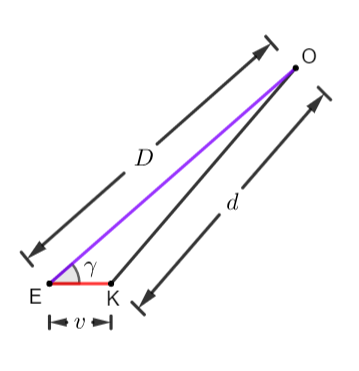
(a)


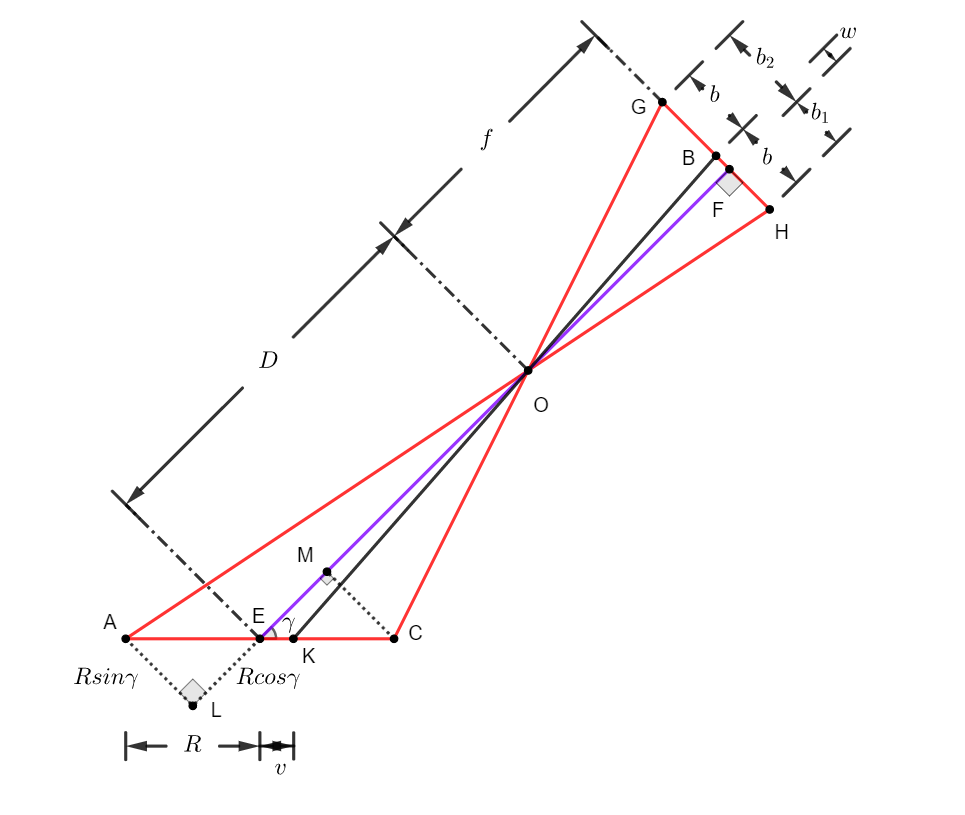

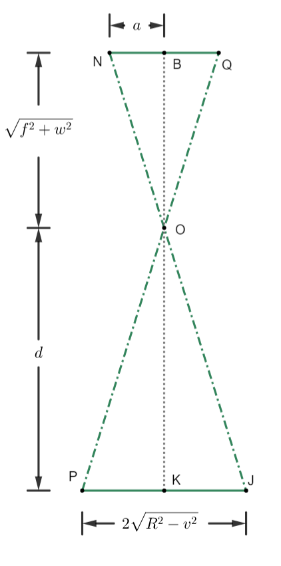


(b) (c)

Figure A1. (a) Perspective relationship between a circular feature and its image (i.e., an ellipse). On the object plane, point E is the center of the circular feature, $\left| \mathrm{AE} \right|$ and $\left| \mathrm{EC} \right|$ are the radius of round plate ($R$).$\angle OEC$= $\gamma$, O is the optical center of the camera,$\left| OE \right|$ is the distance between the optical center and the center of the plate, defined as $D$. $\left| \mathrm{OF} \right|$ is focal length $f.$ On the image plane, H is the projection point of A, G is the projection point of C, and F is the projection point of E, B is the center of the ellipse. $\left| \mathrm{GH} \right|$ is the minor axis of the ellipse. $\left| \mathrm{GB} \right|=\left| \mathrm{BH} \right|=b$,$\left| \mathrm{FH} \right|=b_{1}$ $,\left| \mathrm{GF} \right|=b_{2}$, $\left| \mathrm{BF} \right|=w$. K is the corresponding point of the ellipse center in the object plane. (b) The plane that contains the minor axis of the ellipse and the corresponding radius of the circle. This plane is shown as the red plane in (a). $\left| \mathrm{EL} \right|=\left| \mathrm{EM} \right|=R\cos\gamma$, $\left| \mathrm{AL} \right|=\left| \mathrm{CM} \right|=R\sin\gamma.$ We define $\left| \mathrm{EK} \right|=v$, $\left| \mathrm{KO} \right|=d$.(c) The plane that contains the major axis of the ellipse and the corresponding chord of the circle. $\left| \mathrm{NB} \right|=\left| \mathrm{BQ} \right|=a$. This plane is shown as the green plane in (a).

We plot $1/a$ vs. *D* in the region of *D* and *R* that we are interested (R is from 30 mm to 165 mm), the relationship between $1/a$ and *D* is almost linear if $\gamma$ is constant, see Figure A2 (a). Constant $\gamma$ means that the camera only moves along the optical axis without any rotation, which cannot be guaranteed due to the body movement during eating. Thus $\gamma$ is set to be a random number between 20° and 70° in this simulation. After generating a large number of pairs of ($1/a_{i},D_{i}$)$, i=1,2,\cdots N$ using Eq. (A.12) for different R, we can see that their relationship can still be approximated as linear, examples in Figure A2 (b).


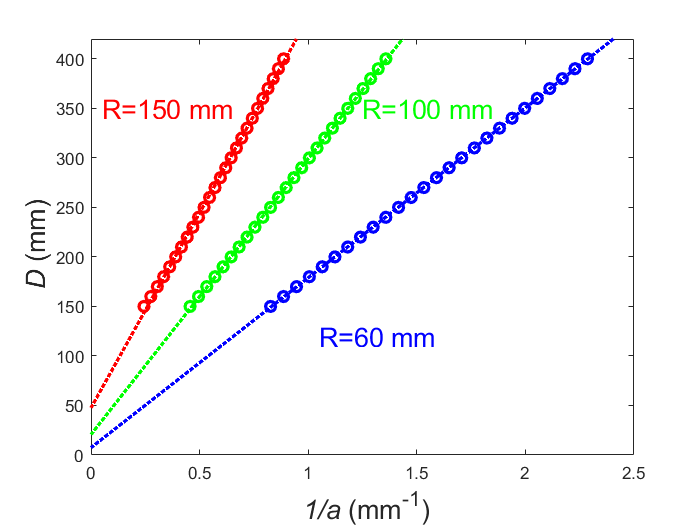

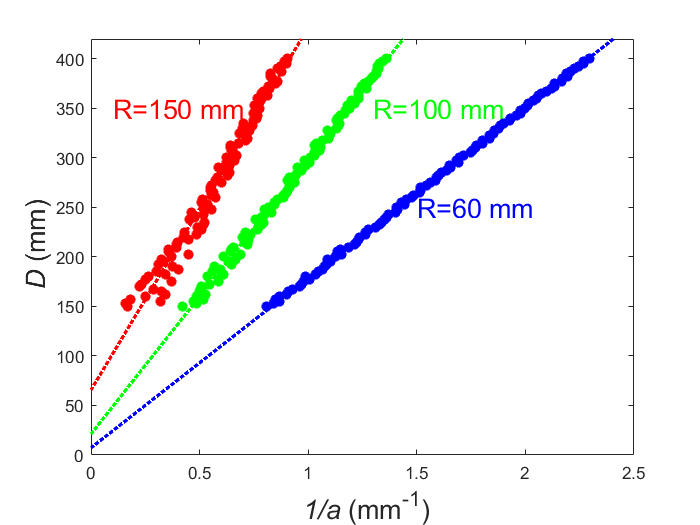


(a) (b)

Figure A2. (a) Simulated camera-to-plate distance vs. 1/*a* using Eq. (A.12) when $\gamma$ is constant; (b) Simulated camera-to-plate distance vs. 1/*a* using Eq. (A.12) when $\gamma$ changes between 20° and 70°.

When $\gamma$ is constant, the relationship between $1/b$ and *D* can also be considered as linear (see Figure A3(a)), however, when $\gamma$ changes, the relationship becomes complicated, shown in Figure A3(b)*.* Thus, we cannot use this relationship to estimate *R* since the effect of $\gamma$ cannot be ignored.


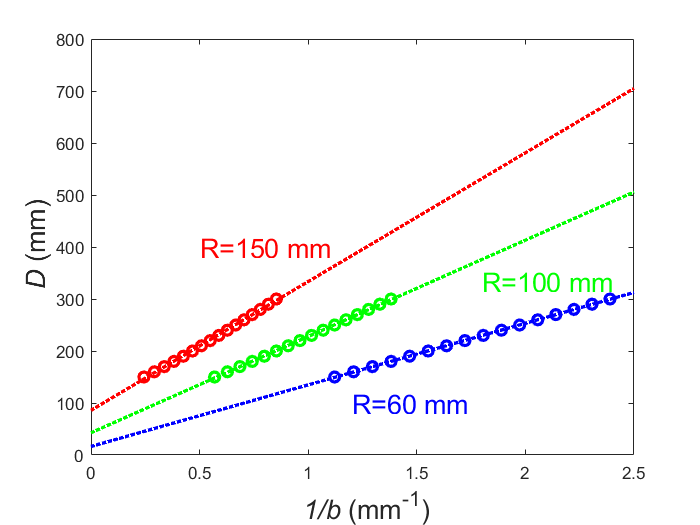

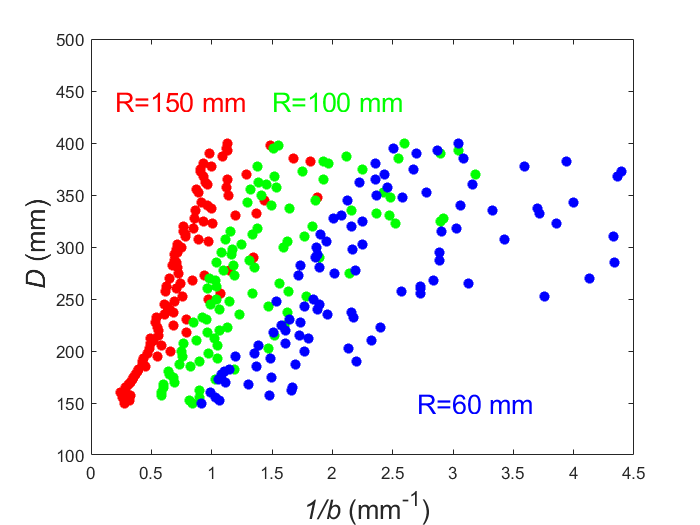


(a) (b)

Figure A3. (a) Simulated camera-to-plate distance vs. 1/*b* using Eq. (A.5) when $\gamma$ is constant; (b) Simulated camera-to-plate distance vs. 1/*b* using Eq. (A.5) when $\gamma$ changes between 20° and 70°.

## Supplements to Section 3.4: Results of the Comparative Experiment

For comparison, we conducted another experiment to estimate the plate radius with a fiducial marker placed on the table. Ten plates with different radii and/or heights were chosen (shown in Table A1 and Figure A4). We utilized the following procedure for plate radius estimation:

1. Detect the fiducial marker (i.e., checkerboard) in each image.
2. Compute a translation vector and a rotation matrix based on the deformation of the fiducial marker in the image. The vector and matrix determine the location and orientation of the camera with respect to the table.
3. Detect the plate border automatically (Nie et al., 2010). In some cases, the automatic method fails due to occlusion, shadowing and various other problems. In this case, six points around the border in the image are manually selected.
4. Adjust the translation vector based on the known or assumed plate height, and project all the points in the image representing the plate border to the real-world coordinate system.
5. Fit a circle using these points after the adjustment. The radius of the circle is the radius of the plate.

For a fair comparison with our method, we assumed that the plate height was standard, which was the height of the reference plate, the same as the assumption made in our method. Under this assumption, each of the ten plates was given a chance to be the reference plate, and the radii of the remaining nine plates were estimated. Thus, total 90 plate radius estimates were obtained. In each estimate, five images captured at different viewing distances and angles were processed using the above procedure, and the outputs were averaged as a single estimate. The estimation results are summarized in Tables A2 and A3.


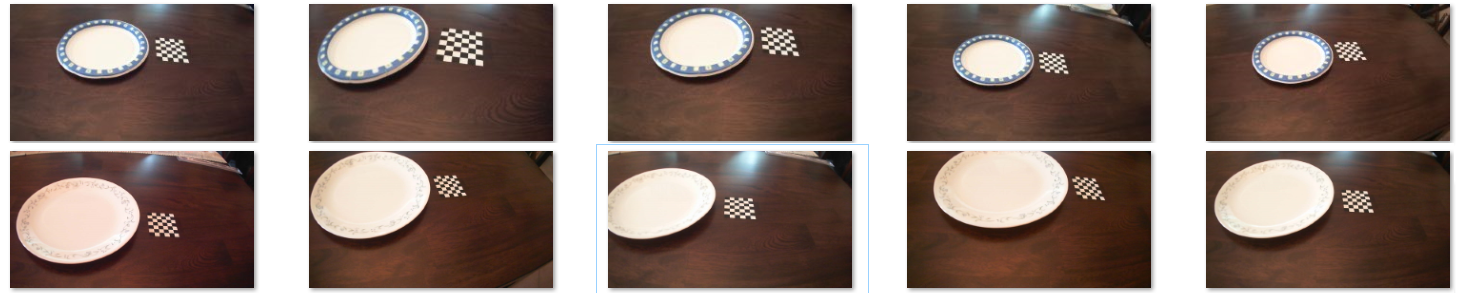
Figure A4. Typical images of plates with a fiducial marker.

Table A1. Measured (ground truth) plate radii and heights for the ten plates.

|  | Plate#1 | Plate#2 | Plate#3 | Plate#4 | Plate#5 | Plate#6 | Plate#7 | Plate#8 | Plate#9 | Plate#10 |
| --- | --- | --- | --- | --- | --- | --- | --- | --- | --- | --- |
| Radius(mm) | 87.5 | 92.5 | 102.5 | 130 | 83.5 | 135 | 110 | 115 | 73.5 | 130 |
| Height(mm) | 18 | 20 | 48 | 20 | 13 | 34 | 38 | 41 | 20 | 24 |

Table A2. Comparison of measured (ground truth) and estimated plate radii for the ten plates. The values in each row (denoted by “Test #”) represent estimated radii of different plates using the same reference (e.g. the boldfaced value), while the values in each column (denoted by “Plate #”) represent estimated radii of the same plate using different references. The boldfaced values along the diagonal lines are true radii, which are actually measured values.

| Radius(mm) | Plate#1 | Plate#2 | Plate#3 | Plate#4 | Plate#5 | Plate#6 | Plate#7 | Plate#8 | Plate#9 | Plate#10 |
| --- | --- | --- | --- | --- | --- | --- | --- | --- | --- | --- |
| Test#1 | **87.5** | 94.7 | 122.1 | 129.3 | 78.3 | 141.4 | 120.1 | 126.9 | 73.0 | 128.0 |
| Test#2 | 85.0 | **92.5** | 121.2 | 128.3 | 77.8 | 140.9 | 119.4 | 126.4 | 72.6 | 127.3 |
| Test#3 | 76.0 | 83.2 | **102.5** | 115.2 | 70.9 | 133.4 | 109.7 | 118.2 | 66.2 | 117.5 |
| Test#4 | 85.0 | 93.9 | 121.2 | **130.0** | 77.8 | 140.9 | 119.4 | 126.4 | 72.6 | 127.3 |
| Test#5 | 87.2 | 96.6 | 124.2 | 131.6 | **83.5** | 142.8 | 121.8 | 128.4 | 74.2 | 129.8 |
| Test#6 | 80.5 | 88.6 | 115.3 | 121.8 | 74.4 | **135.0** | 114.6 | 122.3 | 69.4 | 122.4 |
| Test#7 | 79.2 | 87.1 | 113.6 | 119.9 | 73.4 | 136.1 | **110.0** | 121.1 | 68.5 | 121.0 |
| Test#8 | 78.2 | 85.9 | 112.3 | 118.5 | 72.7 | 135.3 | 112.2 | **115.0** | 67.8 | 119.9 |
| Test#9 | 85.0 | 93.9 | 121.2 | 128.3 | 77.8 | 140.9 | 119.4 | 126.4 | **73.5** | 127.3 |
| Test#10 | 83.7 | 92.4 | 119.5 | 126.5 | 76.8 | 139.8 | 118.0 | 125.2 | 71.7 | **130.0** |

Table A3. Comparison of measured (ground truth) and estimated plate radii for the ten plates by percentage errors. The values in each row (denoted by “Test #”) represent the percentage errors of the estimated radii of different plates using the same reference (e.g. the boldfaced value), while the values in each column (denoted by “Plate #”) represent the percentage errors of the estimated radii of the same plate using different references.

| Percentage Error (%) | Plate#1 | Plate#2 | Plate#3 | Plate#4 | Plate#5 | Plate#6 | Plate#7 | Plate#8 | Plate#9 | Plate#10 |
| --- | --- | --- | --- | --- | --- | --- | --- | --- | --- | --- |
| Test#1 |  | -2.9% | -13.2% | -2.9% | -0.3% | -8.0% | -9.5% | -10.6% | -2.9% | -4.3% |
| Test#2 | 2.4% |  | -10.0% | 1.5% | 4.4% | -4.2% | -5.9% | -7.1% | 1.5% | -0.1% |
| Test#3 | 19.1% | 18.3% |  | 18.3% | 21.2% | 12.5% | 10.8% | 9.5% | 18.3% | 16.6% |
| Test#4 | -0.6% | -1.3% | -11.4% |  | 1.2% | -6.3% | -7.8% | -8.8% | -1.3% | -2.7% |
| Test#5 | -6.3% | -6.9% | -15.0% | -6.9% |  | -10.9% | -12.1% | -13.0% | -6.9% | -8.0% |
| Test#6 | 4.8% | 4.4% | -1.2% | 4.4% | 5.8% |  | 0.8% | 0.2% | 4.4% | 3.6% |
| Test#7 | 9.2% | 8.5% | -0.2% | 8.5% | 10.7% | 4.2% |  | 2.0% | 8.5% | 7.3% |
| Test#8 | 10.4% | 9.9% | 2.7% | 9.9% | 11.7% | 6.3% | 5.3% |  | 9.9% | 8.9% |
| Test#9 | -0.6% | -1.2% | -9.9% | -1.2% | 0.9% | -5.6% | -6.8% | -7.7% |  | -2.5% |
| Test#10 | -1.5% | -2.1% | -9.6% | -2.1% | -0.2% | -5.9% | -6.9% | -7.7% | -2.1% |  |

**References:**

Jia, W., Yue, Y., Fernstrom, J.D., Yao, N., Sclabassi, R.J., Fernstrom, M.H., et al. (2012). Image-based estimation of food volume using circular referents in dietary assessment. *Journal of Food Engineering* 109(1)**,** 76-86.

Nie, J., Wei, Z., Jia, W., Li, L., Fernstrom, J.D., Sclabassi, R.J., et al. (2010). "Automatic detection of dining plates for image-based dietary evaluation", in: *Proc*. *32nd Annual International Conference of the IEEE Engineering in Medicine and Biology Society*, Buenos Aires, Argentina, August 31 - September 4, 4312-4315.

Safaee-Rad, R., Tchoukanov, I., Smith, K.C., and Benhabib, B. (1992). Three-dimensional location estimation of circular features for machine vision. *IEEE Transactions on Robotics and Automation* 8(5)**,** 624-640. doi: 10.1109/70.163786.
